# Robot-Assisted “Postage-Stamp” Vertebrotomy for Spinal Tumor Resection: Case Report

**DOI:** 10.3390/jcm15114268

**Published:** 2026-05-31

**Authors:** Carlo Brembilla, Gabriele Capo, Mario De Robertis, Umberto Cariboni, Ali Baram, Donato Creatura, Emanuele Stucchi, Leonardo Anselmi, Federico Pessina, Maurizio Fornari

**Affiliations:** 1 Department of Neurosurgery, IRCSS Humanitas Research Hospital, Via Manzoni 56, Rozzano, 20089 Milan, Italy; carlo.brembilla@humanitas.it (C.B.); mario.derobertis@humanitas.it (M.D.R.); ali.baram@humanitas.it (A.B.); donato.creatura@humanitas.it (D.C.);; 2 Department of Biomedical Sciences, Humanitas University, Via Rita Levi Montalcini 4, Pieve Emanuele, 20090 Milan, Italy; 3 Department of Thoracic Surgery, IRCSS Humanitas Research Hospital, Via Manzoni 56, Rozzano, 20089 Milan, Italy; umberto.cariboni@cancercenter.humanitas.it

**Keywords:** chondrosarcoma, spinal tumors, robotics, image-guided surgery, osteotomy, thoracic vertebrae

## Abstract

**Background**: Achieving adequate oncological margins in tumors involving the thoracic costovertebral junction is technically challenging because of complex regional anatomy and the need to preserve neurological and biomechanical integrity. This case report describes a robot-assisted margin-extension strategy after incomplete resection of a thoracic costovertebral chondrosarcoma. **Methods**: A 31-year-old man with grade 1 chondrosarcoma of the left sixth rib underwent second-stage surgical radicalization after prior incomplete resection with positive medial margins. Following multidisciplinary discussion, a single-stage posterior procedure was performed, including robot-assisted T4–T8 stabilization with radiolucent CFR-PEEK instrumentation and robot-assisted sagittal vertebral osteotomy (“Postage-Stamp Osteotomy”) of T6 to achieve en bloc removal of the involved costovertebral segment. **Results**: The osteotomy was executed using a pedicle-referenced robotic trajectory workflow with sequential navigated drilling and controlled completion with a navigated osteotome. Total operative time was 379 min, with estimated blood loss of 800 mL. No major intraoperative neurovascular complications occurred. Histopathology confirmed negative margins. The patient was mobilized on postoperative day 1 and discharged on postoperative day 6 without new neurological deficits. Radiological follow-up at 3 months showed no recurrence, while clinical follow-up at 5 months demonstrated full return to baseline activities. **Conclusions**: This report describes a technically feasible robot-assisted margin-extension strategy in a highly selected thoracic spinal oncology scenario. Although long-term oncological conclusions cannot be drawn from a single case, tailored technology-enabled margin-oriented approaches may represent a case-specific option in carefully selected patients.

## 1. Introduction

Advances in spinal oncology surgery have progressively shifted practice from standardized, highly morbid resections to patient-specific, margin-oriented strategies aimed at balancing oncological adequacy with neurological preservation and biomechanical stability. In this setting, intraoperative three-dimensional imaging, navigation, and robotic guidance have improved the accuracy and reproducibility of complex spinal procedures, facilitating the translation of preoperative planning into controlled osteotomies in anatomically constrained regions [1,2,3,4,5,6].

Radiolucent spinal instrumentation, including carbon fiber–reinforced polyetheretherketone (CFR-PEEK) systems, further supports oncological surgery by reducing the CT and MRI artifact burden compared with conventional metallic implants, thereby improving postoperative assessment and oncological surveillance and facilitating radiotherapy planning when adjuvant treatment is required [7,8,9]. Modern guidance technologies and radiolucent implants have expanded the feasibility of tailored resections in selected oncological scenarios.

Chondrosarcomas involving the thoracic spine and costovertebral junction remain challenging. Chondrosarcoma is the second most common primary malignant bone tumor in adults and frequently occurs in the chest wall, particularly in the ribs, with locally aggressive behavior and proximity to critical neurovascular structures [10,11]. Given their resistance to chemotherapy and radiotherapy, surgical resection with adequate margins remains the cornerstone of treatment, especially in low-grade lesions, where local control is primarily driven by the margin status [11,12,13,14,15].

When the disease extends to the costovertebral junction or vertebral body, oncological radicality may require complex procedures, including partial vertebral resection or en bloc spondylectomy [16,17,18]. In highly selected low-grade tumors with limited vertebral involvement, case-specific margin-oriented strategies aimed at extending oncological margins, rather than pursuing radical en bloc resection, may occasionally be considered when supported by contemporary enabling technologies and when the anticipated morbidity of more extensive procedures is substantial [19,20,21,22,23].

Here, we report a case of grade 1 chondrosarcoma of the left sixth rib that required second-stage surgical radicalization after incomplete initial resection. The procedure consisted of oncological margin extension through a robot-assisted sagittal vertebral osteotomy (“Postage-Stamp Osteotomy”) combined with posterior spinal stabilization using CFR-PEEK instrumentation. This case report describes the planning and stepwise execution of the robot-assisted vertebral split for oncological margin extension within the emerging experience of robotic assistance in complex spinal oncology procedures [24,25,26].

## 2. Clinical Presentation

A 31-year-old man was referred to the Thoracic Surgery Unit after a single episode of spontaneous hemothorax. Imaging studies revealed a mass involving the left sixth rib with extension toward the costovertebral junction (Figure 1). Percutaneous needle biopsy performed in October 2025 established a diagnosis of grade 1 chondrosarcoma.

The patient underwent surgical resection of the affected rib through left posterior thoracotomy. Histopathological examination confirmed low-grade chondrosarcoma; however, the medial surgical margin was positive, with residual disease identified at the level of the rib head (Figure 2).

Following multidisciplinary discussions within the institutional Rare Tumor Board (sarcoma and rare tumors), surgical margin extension was recommended. Given the involvement of the costovertebral junction and the adjacent vertebral body, a combined multidisciplinary surgical strategy was planned, with close collaboration between the thoracic and neurosurgical teams for further management.

A single-stage posterior approach was planned, including posterior spinal stabilization from T4 to T8 using CFR-PEEK instrumentation combined with targeted resection of the costovertebral and vertebral structures involved in oncological margin extension. Owing to the need for precise execution in close proximity to the neural elements, the procedure was planned and performed using robotic navigation.

The postoperative course was overall favorable, although not entirely uneventful. The patient was admitted to the intensive care unit for overnight postoperative monitoring and transferred to the neurosurgical ward on postoperative day 1. No new neurological deficits or new radicular pain occurred following surgery. Pain was satisfactorily controlled with scheduled analgesic therapy, allowing early mobilization with spinal orthosis and autonomous ambulation from postoperative day 1. Total operative time was 379 min, with an estimated intraoperative blood loss of approximately 800 mL. During hospitalization, postoperative anemia required transfusion of two units of packed red blood cells.

A transient episode of oxygen desaturation prompted thoracic imaging, which did not reveal acute complications requiring intervention. Beginning on postoperative day 3, the patient experienced sporadic mild hemoptysis, which progressively resolved with conservative management. Thoracic CT demonstrated no active bleeding and no pneumothorax. The patient was discharged on postoperative day 6 in stable clinical condition, eupneic in room air, independently ambulatory, with satisfactory pain control and no new neurological deficits.

Postoperative computed tomography confirmed adequate resection and correct instrumentation placement. The resected specimen measured 5.2 cm × 3.4 cm × 2.3 cm and included vertebral bone, the costovertebral articulation, and surrounding soft tissues. Gross pathological examination identified residual translucent brownish tissue corresponding to focal residual chondrogenic proliferation, approaching the left lateral margin to approximately 2 mm on macroscopic assessment. The specimen was oriented for pathological evaluation, with separate assessment of superior, inferior, right lateral, left lateral, and anterior planes (the anterior surface being inked for orientation). Histological examination confirmed focal residual chondrogenic proliferation consistent with the previously diagnosed low-grade chondrosarcoma, within a background of extensive post-surgical inflammatory and reparative changes. Final pathological assessment confirmed negative excision margins. Accordingly, no adjuvant therapy was indicated.

At the 3-month follow-up, photon-counting CT confirmed the stability of the construct and previously documented extent of resection (Figure 3). At the 5-month clinical follow-up, the patient had returned to baseline daily, occupational, and physical activities, with only expected persistent hypoesthesia in the thoracic sensory territory corresponding to the sacrificed nerve root.

## 3. Surgical Technique

### 3.1. Patient Positioning and Intraoperative Setup

The procedure was performed under general anesthesia, with the patient in the prone position on a radiolucent spinal table. Continuous intraoperative neurophysiological monitoring, including somatosensory and motor-evoked potentials, was performed throughout the procedure. An operating microscope is employed during the microsurgical steps to provide high-magnification visualization of the neural and adjacent anatomical structures.

Intraoperative three-dimensional imaging was performed using an O-arm™ imaging system (O2 imaging software version 4.2.1; Medtronic, Minneapolis, MN, USA) and registered on a robotic navigation platform (Excelsius GPS^®^ with gmed^®^ software version 6.1; Globus Medical, Audubon, PA, USA). The robotic system was used for preoperative planning, trajectory definition, and real-time intraoperative guidance for both spinal instrumentation and vertebral osteotomy steps, allowing for accurate execution of predefined trajectories and depth-controlled bone resection.

### 3.2. Posterior Exposure and Instrumentation

A standard posterior midline approach was used, followed by subperiosteal exposure of the posterior elements across the planned stabilization levels.

Posterior fixation was performed from T4 to T8 using robot-assisted pedicle screw placement, according to the preplanned trajectories. All the screws were inserted under robotic guidance to ensure accurate alignment and depth control. At T6, the pedicle screw was placed only on the right side, whereas instrumentation of the tumor-involved pedicle was intentionally omitted to avoid violation of the lesion.

Radiolucent CFR-PEEK pedicle screw instrumentation (VADER^®^ Pedicle System; Icotec AG, Altstätten, Switzerland) was used to facilitate the postoperative imaging assessment, oncological surveillance, and potential radiotherapy planning if adjuvant treatment had become necessary. Screw trajectories were robotically planned according to the implant geometry and regional anatomy. A temporary right-sided rod was applied after screw placement to ensure construct stability during tumor resection.

### 3.3. Posterior Decompression and Neural Exposure

Complete laminectomy was performed at T5 and T6, including the superior margin of the T7 lamina, allowing full exposure of the dural sac. Complete left T5–T6 and T6–T7 facetectomies were then performed to expose the exiting left T5 and T6 nerve roots. The roots were identified, coagulated, and transected to allow for oncological resection. This step provided sufficient neural decompression and established a safe operative corridor for the subsequent tumor resection phase.

### 3.4. Lateral Costovertebral Margin Definition

First, the lateral margin of the vertebrocostal segment to be resected was defined. Limited lateral exposure of the thoracic cage was performed, superiorly identifying the inferior border of the fifth rib and inferiorly identifying the superior border of the seventh rib. The previous proximal resection margin of the sixth rib was carefully dissected and preserved as it is known to be oncologically free. This step allowed the circumferential delineation of the vertebrocostal segment to be removed and established a safe lateral oncological boundary before proceeding with vertebral osteotomy.

#### Pedicle Preparation and Osteotomy Plane Identification

The posteromedial aspect of the left T6 pedicle was identified and selectively drilled along the medial portion while preserving the lateral cortex. The residual lateral pedicle defined the lateral limit of resection, representing an oncologically safe margin, and provided a reproducible osseous guide for planning sagittal osteotomy. This step was essential to achieve an adequate margin along the posterior aspect of the T6 vertebral body while working within a very narrow corridor between the intended resection volume and dural sac and spinal cord.

### 3.5. Robot-Assisted “Postage-Stamp” Vertebral Osteotomy (Figure 4)

Using robotic navigation, sagittal osteotomy was planned based on a previously created medial pedicle reference. The planned resection included the left lateral third of the T6 vertebral body and the inferoposterior portion of the T5 vertebral body corresponding to the cranial extent of the sixth left costovertebral articulation.

Four navigated drill tracts were created along a predefined trajectory in the planned osteotomy plane within the vertebral body. These tracts were preoperatively planned on the robotic platform using a screw-based planning workflow (4 mm × 30 mm virtual trajectories). Each tract was advanced under navigation control to a predetermined depth, ensuring accuracy and preventing violations of the contralateral cortex or adjacent neural structures.

The inferior limit of the osteotomy corresponded to the T6–T7 intervertebral disc, whereas the superior limit included the inferoposterior portion of the T5 vertebral body, thereby ensuring an oncologically adequate cranial margin.

This stepwise drilling pattern produced progressive weakening of the vertebral body along the intended sagittal plane, resembling a “postage-stamp” configuration. Individual drill tracts were subsequently connected using a navigated osteotome to complete the sagittal vertebral split in a controlled and reproducible manner.

The robot-assisted, depth-controlled drilling strategy allowed for precise modulation of the osteotomy trajectory despite the constrained surgical corridor, maintaining constant spatial awareness and minimizing the risk of unintended cortical breach or neural injury.

**Figure 4:**
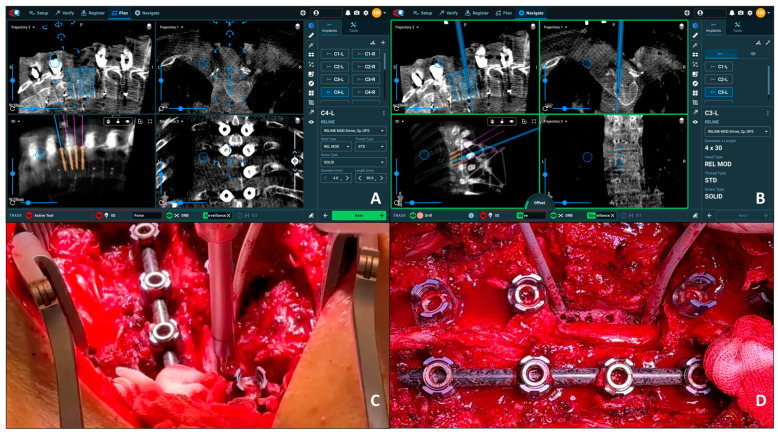
**Robot-assisted “postage-stamp” vertebral osteotomy: planning and execution of drill trajectories**. Intraoperative and robotic planning images illustrating the stepwise creation of navigation-guided drill tracts forming the sagittal osteotomy plane. (**A**) Preoperative robotic planning screenshot showing four parallel trajectories within the left lateral third of the T6 vertebral body, corresponding to the planned “postage-stamp” osteotomy configuration. (**B**) Intraoperative robotic navigation screenshot demonstrating real-time execution of a single drill tract along the predefined trajectory under robotic guidance. (**C**) Intraoperative photograph showing the high-speed drill mounted within the robotic arm holder during trajectory execution. (**D**) Intraoperative view of the vertebral body after completion of the four drill tracts along the left lateral margin, illustrating the characteristic “postage-stamp” configuration defining the planned sagittal vertebral split.

### 3.6. En Bloc Oncological Resection

Completion of navigated drilling and osteotome cuts allowed mobilization of the planned costovertebral segment (Figure 5). Progressive lateralization maneuvers were performed to displace the specimen, and the external cortical margins on the thoracic side of the vertebral body were refined using the Kerrison rongeurs.

Once fully mobilized, the specimen remained attached only along the anterolateral aspect. At this stage, dense post-surgical adhesions were encountered between the specimen, the pleural plane, and adjacent mediastinal structures, partly related to the previous thoracic surgery. Progressive blunt dissection was initially performed to identify and develop residual tissue planes, followed by careful sharp dissection in areas of particularly tenacious adhesions. The descending thoracic aorta was progressively identified under direct visualization, meticulously protected, and completely freed during the final stages of mobilization, remaining intact and pulsatile at the conclusion of the procedure.

Following complete release of the residual anterolateral attachment, the specimen was removed en bloc (Figure 6).

The operative cavity remained functionally separated from the pleural space by dense cicatricial tissue resulting from the previous thoracotomy. During adhesiolysis, only limited focal pleural breaches occurred, which were immediately repaired by the thoracic surgeon using primary suturing reinforced with a collagen patch (TachoSil^®^; Takeda Pharmaceuticals International AG, Zurich, Switzerland) and fibrin sealant. Because no formal communication with the pleural cavity persisted, pleural drainage was not required. A standard posterior surgical wound drain was placed in the operative cavity.

### 3.7. Posterior Reconstruction and Arthrodesis

Definitive posterior stabilization was performed by securing the rods bilaterally to the pedicle screws. The contralateral (right-sided) facet joints of the instrument levels were decorticated to prepare the arthrodesis bed. Posterolateral fusion was only performed on the contralateral side. A synthetic bone graft substitute (bioactive glass, NovaBone^®^; NovaBone Products, Alachua, FL, USA) was used to promote fusion. Final tightening confirmed the construct stability.

Intraoperative neuromonitoring remained stable throughout the procedure. Final three-dimensional imaging confirmed the adequate extent of resection and correct implant positioning. The wound was closed in anatomical layers over the surgical drains.

## 4. Discussion

Chondrosarcomas involving the costovertebral junction with extension into the vertebral body represent a rare oncological scenario for which standardized surgical strategies are lacking. Chondrosarcoma is the second most common primary malignant bone tumor in adults and frequently involves the chest wall, particularly the ribs, where it poses significant surgical challenges owing to its local aggressiveness and proximity to vital structures [10,14,27]. Although conventional low-grade (grade 1) chondrosarcomas are slow growing, they demonstrate a marked propensity for local recurrence when inadequately treated. Given their well-documented resistance to chemotherapy and radiotherapy, surgical resection with wide negative margins (R0) remains the cornerstone of treatment [12,15]. While wide en bloc resection is considered the oncological ideal, the thoracic spine presents unique anatomical, neurological, and biomechanical constraints that frequently preclude true en bloc excision, particularly at the costovertebral junction [13,14,17]. This challenge is especially relevant in low-grade lesions, where oncological radicality must be carefully balanced against neurological risk and postoperative functional outcomes [13]. The reported 5-year survival rates following complete resection range from 60% to >90%, whereas incomplete resections are associated with significantly higher rates of local recurrence and disease-related mortality [28].

The present case introduces a technically distinctive application of robotic navigation in oncological spinal surgery, extending its role beyond instrumentation accuracy to robot-guided bone cutting and margin-oriented vertebral osteotomy planning and execution. Tailored vertebral osteotomies have previously been described in complex oncological resections to adapt resection planes to tumor extension while preserving critical structures, such as the spinal canal. Gasbarrini et al., for example, reported an “L-shaped” osteotomy allowing for en bloc resection of a thoracic chordoma while maintaining canal continuity and achieving oncologically appropriate margins [29]. Robotic platforms have been increasingly adopted in spine surgery, demonstrating high precision and safety in pedicle screw placement, even in complex anatomical and oncological scenarios [2,3,30,31]. However, their application in oncological bone cuts remains far less explored. Recent reviews have highlighted the emerging role of robotic platforms in spinal oncology, particularly for complex resections requiring precise trajectory planning and reproducible execution of technically demanding surgical steps. Isolated reports have described the use of robotic guidance to plan and execute vertebral osteotomies during oncologic resections [32]. More recently, robotic assistance has been explored for tumor-related spinal procedures, including complex osteotomies and oncological resections [20,29,33].

To our knowledge, this represents one of the first detailed reports describing the use of a robotic platform to plan and execute a sagittal vertebral body division specifically aimed at oncological margin extension following prior incomplete resection of a thoracic costovertebral chondrosarcoma. While previous studies have highlighted the utility of robotic guidance for instrumentation, puncture guidance, and selected tumor resections, detailed descriptions of margin-oriented robot-assisted vertebral osteotomy workflows remain limited in spinal oncology.

In the current case, robotic guidance allowed for precise definition and intraoperative reproduction of the planned osteotomy plane within the T6 vertebral body. Similar concepts have been reported in isolated cases where robotic systems were used to guide vertebral osteotomies during tumor resections, supporting the feasibility of robot-guided bone-cutting trajectories in spinal oncology [6]. This level of accuracy was essential to achieving medial margin extension beyond the previously positive rib head margin while preserving the contralateral vertebral body, spinal canal, and segmental stability.

We refer to this incremental, navigation-guided perforation strategy as the “Postage-Stamp Osteotomy.” Its conceptual novelty does not lie in any single isolated technical element but rather in the integration of several components within a specific oncological application: robot-assisted trajectory planning adapted from screw-based workflows, sequential depth-controlled navigated drill tracts along a predefined sagittal osteotomy plane, controlled completion of the vertebral division using a navigated osteotome, and the application of this workflow specifically for oncological margin extension after prior incomplete resection.

The adoption of descriptive terminology for specific osteotomy configurations has precedent in spinal oncology. Gasbarrini et al., for example, described an “L-shaped” osteotomy designed to adapt the resection plane to tumor extension while preserving the spinal canal during en bloc resection of a thoracic chordoma [29]. In contrast to a predefined geometric osteotomy such as the L-shaped configuration, the “Postage-Stamp Osteotomy” described in the present report relies on sequential navigation-guided perforations that allow the surgeon to progressively construct the osteotomy line while maintaining continuous control of the trajectory relative to tumor margins and adjacent neural structures. This incremental strategy may be particularly advantageous in anatomically constrained regions of the spine, where the distance between the tumor margin and the spinal canal may be limited to only a few millimeters, and precise trajectory control becomes critical for preserving neural structures while achieving oncological margin extension.

The “postage-stamp” concept has been used in other surgical fields to describe controlled, sequential perforations along a planned osteotomy line, particularly in facial plastic surgery [34,35,36].

Compared with more extensive procedures such as en bloc spondylectomy, which, although oncologically sound, are associated with substantial morbidity and neurological risk [17,18], the vertebral split approach described in this case may represent a highly selected case-specific margin-oriented alternative when the anticipated morbidity of more extensive procedures is considerable. This tailored strategy may be particularly relevant in selected low-grade chondrosarcomas, where local control is primarily determined by surgical margins rather than adjuvant therapies.

The use of CFR-PEEK instrumentation represented an intentional oncological adjunct in this case rather than a purely reconstructive choice. Compared with conventional metallic implants, CFR-PEEK systems substantially reduce CT and MRI artifact burden, thereby improving postoperative assessment of resection margins, facilitating surveillance for local recurrence, and optimizing radiotherapy planning should adjuvant treatment become necessary [7,8,9,37,38,39]. Although adjuvant radiotherapy was ultimately not indicated in the present case because final pathology confirmed negative margins in a low-grade lesion, this material characteristic remains particularly relevant in spinal oncology, where postoperative imaging interpretation and multidisciplinary treatment planning are often strongly influenced by implant-related artifacts.

This report also illustrates the broader evolution of spinal robotics from instrumentation guidance toward more versatile intraoperative applications. Robotic platforms were initially introduced to improve pedicle screw placement accuracy and reduce radiation exposure but are increasingly being explored for more complex surgical workflows, including biopsies, tumor access planning, and navigated osteotomies [2,3,6,30,31,32,33]. The present case supports this evolving paradigm by demonstrating the feasibility of adapting a screw-planning robotic workflow to controlled oncological bone cutting in a highly constrained thoracic environment.

However, the present report should be interpreted within the inherent limitations of a single-case experience. The absence of standardized prospective patient-reported outcome measures and the retrospective nature of perioperative data collection limit the granularity of outcome interpretation. Although the pathology report documented negative excision margins, including a closest reported margin of approximately 2 mm on gross assessment, a detailed microscopic clearance map for each individual margin was not available. In addition, the limited duration of oncological radiological follow-up precludes any conclusions regarding long-term local control, recurrence risk, or broader reproducibility. While the described technical workflow proved effective in this individual patient, it should not be interpreted as a generalizable alternative to established oncological resection strategies.

Furthermore, this case reflects a highly specific anatomical and oncological scenario: a low-grade thoracic chondrosarcoma requiring second-stage medial margin extension after prior incomplete rib resection. Accordingly, extrapolation to other tumor histologies, spinal levels, or more extensive vertebral involvement should be approached with caution.

Nevertheless, the present case suggests that in highly selected circumstances, contemporary enabling technologies may expand the technical feasibility of tailored margin-oriented resections that would otherwise require substantially more morbid procedures. Future applications of robotic navigation in spinal oncology may further broaden as software integration, instrument compatibility, and workflow flexibility continue to evolve.

## 5. Conclusions

This case report describes a technically feasible robot-assisted margin-extension strategy for a highly selected thoracic spinal oncology scenario involving prior incomplete resection of a low-grade costovertebral chondrosarcoma with limited vertebral involvement.

The described workflow combines robot-assisted trajectory planning, sequential depth-controlled navigated drilling, and controlled sagittal vertebral osteotomy (“Postage-Stamp Osteotomy”) to achieve oncological margin extension within a constrained anatomical corridor while preserving critical neural and biomechanical structures.

In this individual case, the procedure allowed for the successful completion of the planned resection with negative pathological margins without major intraoperative neurovascular complications or postoperative neurological deterioration.

However, as a single-case experience with limited oncological follow-up, this report does not allow for conclusions regarding long-term local control, recurrence risk, survival outcomes, or broader reproducibility.

Rather than proposing a generalized alternative to established oncological strategies such as en bloc spondylectomy, this experience suggests that highly tailored, technology-enabled margin-oriented approaches may occasionally be considered in carefully selected cases where the anticipated morbidity of more extensive resection is substantial.

Further clinical experience will be required to define the reproducibility, indications, and oncological role of such approaches in spinal tumor surgery.

## Figures and Tables

**Figure 1:**
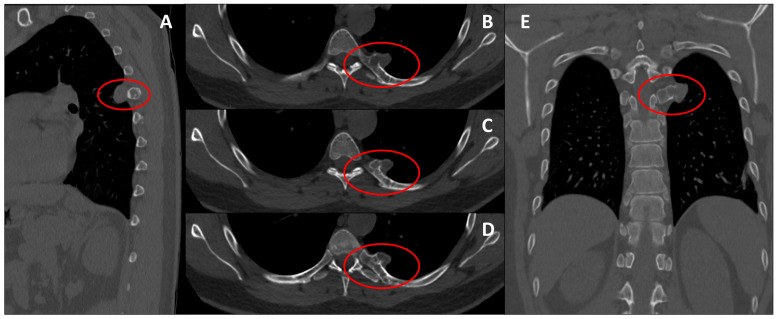
**Initial diagnostic CT scan of the lesion.** Initial diagnostic CT demonstrating a lytic lesion arising from the left sixth rib prior to biopsy. All images are displayed in bone window settings. The lesion originates from and involves the sixth rib, with an exophytic endothoracic component extending up to 21 mm in maximum dimension. The lesion is highlighted with a red circle in all panels. (**A**) Sagittal view; (**B**–**D**) axial views; (**E**) coronal view.

**Figure 2:**
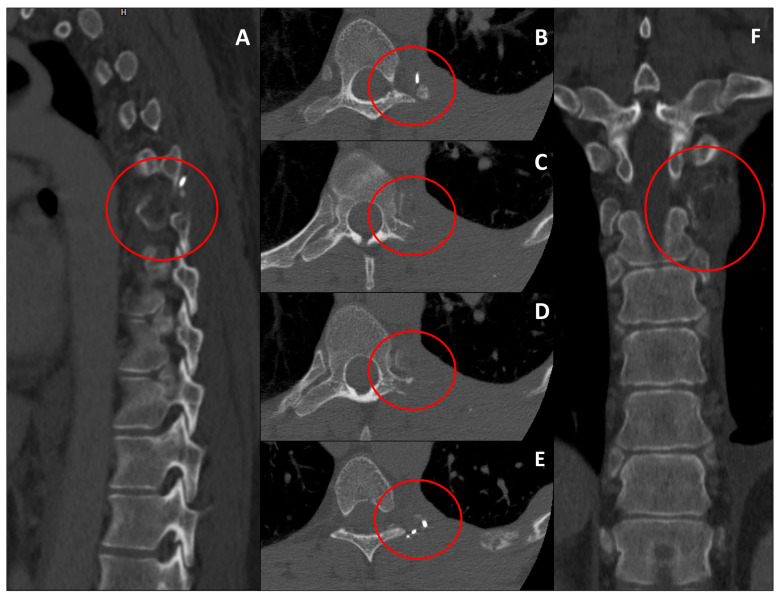
**Postoperative CT after initial thoracic surgery.** Postoperative CT scan performed 40 days after the initial thoracic surgery, following histopathological confirmation of a positive medial margin. All images are displayed in bone window settings. The study demonstrates the residual rib head at the left sixth costovertebral junction, corresponding to the site of residual disease and serving as the anatomical target for subsequent vertebral margin extension. This CT served as the basis for planning the second-stage oncological procedure. The residual rib head is highlighted with red circles in all panels. (**A**) Sagittal view; (**B**–**E**) sequential axial views in caudo-cranial direction; (**F**) coronal view.

**Figure 3:**
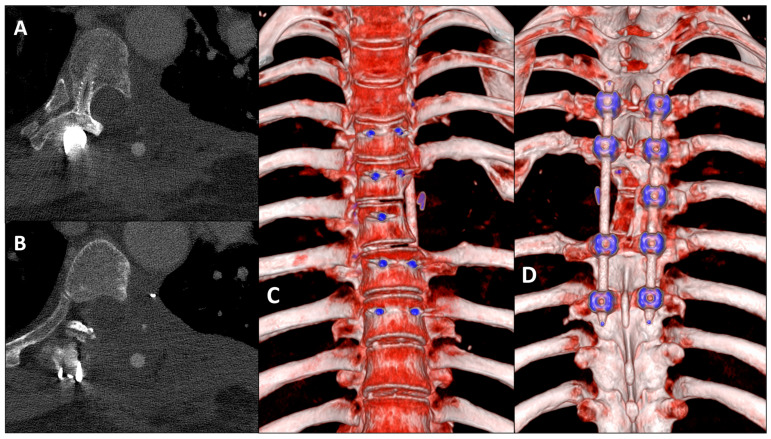
Postoperative oncological follow-up imaging (3-month photon-counting CT). Postoperative photon-counting CT performed at the 3-month oncological follow-up after robot-assisted margin extension. Images are displayed using bone window settings and three-dimensional reconstructions. The study demonstrates the left lateral sagittal vertebral split at T6 (“Postage-Stamp Osteotomy”), confirming adequate margin extension at the level of the left sixth costovertebral junction, with no radiological evidence of residual disease, as well as stable construct alignment with correctly positioned CFR-PEEK instrumentation from T4 to T8 and early signs of posterolateral fusion on the contralateral (right) side. (**A**,**B**) Axial bone-window CT images showing the vertebral split. (**C**) Three-dimensional reconstruction, anteroposterior view. (**D**) Three-dimensional reconstruction, posteroanterior view.

**Figure 5:**
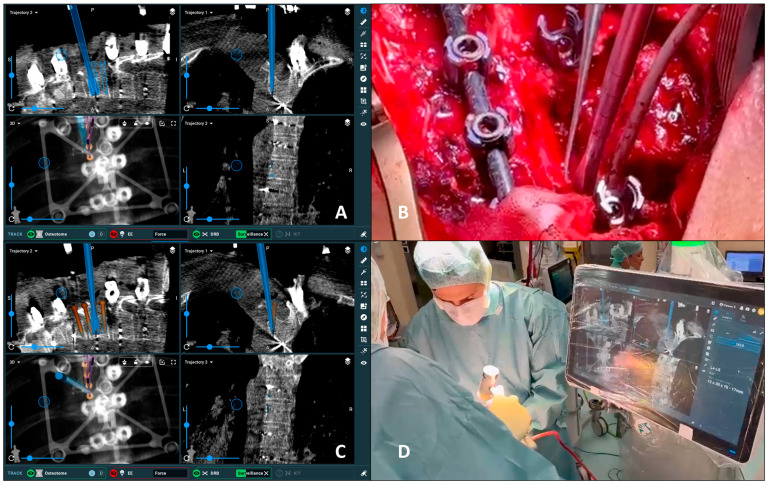
**Robot-assisted completion of the “postage-stamp” vertebral osteotomy using a navigated osteotome.** Intraoperative and navigation images illustrating the connection of the previously created drill tracts and completion of the sagittal vertebral split. (**A**) Intraoperative navigation screenshot demonstrating the advancement of a navigated osteotome through adjacent drill tracts within the left lateral third of the T6 vertebral body, following the predefined osteotomy plane. (**B**) Intraoperative photograph showing the osteotome positioned along the planned trajectory, corresponding to the navigation view in panel (**A**). (**C**) Navigation screenshot depicting the osteotome approaching the distal cortical margin, where controlled lateralization maneuvers are performed to complete the osteotomy and initiate progressive mobilization of the specimen. (**D**) Intraoperative view of the surgical team performing osteotome maneuvers under continuous navigation guidance, with real-time visualization on the navigation monitor.

**Figure 6:**
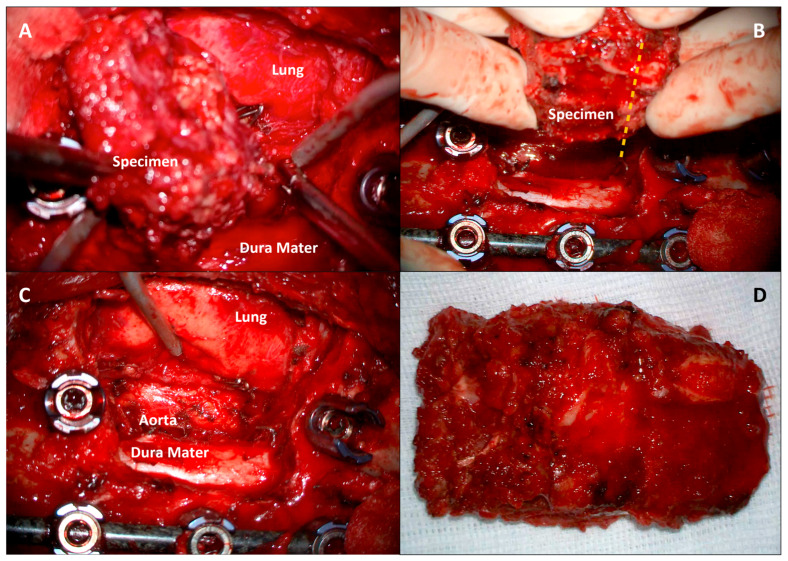
**En bloc resection of the costovertebral specimen and final surgical field.** Microscope-assisted intraoperative views illustrating the final stages of specimen mobilization, en bloc removal, and post-resection anatomy. (**A**) Final phase of specimen mobilization following completion of the sagittal vertebral split. The dural sac is visible in the foreground, while the underlying lung parenchyma becomes evident in the depth of the surgical field. (**B**) Resected specimen immediately after en bloc removal, with the medial vertebral margin exposed. A dashed line indicates the intervertebral disc plane between adjacent vertebral bodies. (**C**) Surgical cavity after specimen removal, demonstrating preservation of critical structures, including the dural sac, descending aorta, and lung. (**D**) Gross specimen placed on surgical gauze, oriented with the medial vertebral surface facing downward, thereby exposing the lateral vertebral margin and the residual rib head segment included within the resection.

## Data Availability

The original contributions presented in this study are included in the article material. Further inquiries can be directed to the corresponding author.
